# 2-Acetyl­pyridinium 3-amino-2-chloro­pyridinium tetra­chloridocobaltate(II)

**DOI:** 10.1107/S1600536809000713

**Published:** 2009-01-10

**Authors:** Ariel Adamski, Violetta Patroniak, Maciej Kubicki

**Affiliations:** a Department of Chemistry, Adam Mickiewicz University, Grunwaldzka 6, 60-780 Poznań, Poland

## Abstract

In the title complex, (C_5_H_6_ClN_2_)(C_7_H_8_NO)[CoCl_4_], the Co^II^ ions are tetra­hedrally coordinated. The crystal structure is built from hydrogen-bonded centrosymmetric tetra­mers of tetra­chloridocobaltate(II) dianions and 3-amino-2-chloro­pyridinium cations, additionally strengthened by significant π–π stacking of pyridinium rings [interplanar distance 3.389 (3) Å]. The tetra­mers are linked by N—H⋯Cl hydrogen bonds into chains; the second kind of cations, *viz.* 2-acetyl­pyridinium, are connected by N—H⋯Cl hydrogen bonds to both sides of the chain. The Co—Cl bond lengths in the dianion correlate with the number of hydrogen bonds accepted by the Cl atom. An intramolecular C—H⋯Cl interaction is also present.

## Related literature

There are only few examples of structures involving the ligands present in the title structure. For related structures, see: 2-acetyl­pyridine itself (Laurent, 1966 ▶) and its cation in perchlorate (Husak, 1996 ▶) and in the complex with tetra­phenyl­porphyrin-zinc(II) (Byrn *et al.*, 1993 ▶), and a free base 3-amino-2-chloro­pyridine (Saha *et al.*, 2006 ▶), and the latter as the dihydrogenphosphate (Hamed *et al.*, 2007 ▶) and as the silver complexes (Tong *et al.*, 2002 ▶; Li *et al.*, 2002 ▶). For literature on the Schiff base complexes, see Häner & Hall (1999 ▶); Mukherjee *et al.* (2005 ▶); Radecka-Paryzek *et al.* (2005 ▶); Yam & Lo (1999 ▶).
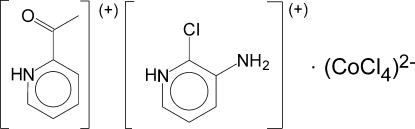


## Experimental

### 

#### Crystal data

(C_5_H_6_ClN_2_)(C_7_H_8_NO)[CoCl_4_]
*M* *_r_* = 452.44
Triclinic, 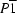

*a* = 7.3255 (5) Å
*b* = 8.3188 (5) Å
*c* = 16.2657 (11) Å
α = 89.114 (5)°
β = 82.806 (5)°
γ = 64.145 (6)°
*V* = 884.13 (10) Å^3^
*Z* = 2
Mo *K*α radiation
μ = 1.73 mm^−1^
*T* = 100 (1) K
0.4 × 0.15 × 0.1 mm

#### Data collection

Kuma KM-4-CCD four-circle diffractometer
Absorption correction: multi-scan (*CrysAlis RED*; Oxford Diffraction, 2007 ▶) *T*_min_ = 0.616, *T*_max_ = 0.841
10954 measured reflections
3798 independent reflections
3470 reflections with *I* > 2σ(*I*)
*R*_int_ = 0.019

#### Refinement

*R*[*F* ^2^ > 2σ(*F* ^2^)] = 0.031
*wR*(*F* ^2^) = 0.065
*S* = 1.24
3798 reflections
216 parameters
H atoms treated by a mixture of independent and constrained refinement
Δρ_max_ = 0.70 e Å^−3^
Δρ_min_ = −0.36 e Å^−3^

### 

Data collection: *CrysAlis CCD* (Oxford Diffraction, 2007 ▶); cell refinement: *CrysAlis RED* (Oxford Diffraction, 2007 ▶); data reduction: *CrysAlis RED*; program(s) used to solve structure: *SHELXS97* (Sheldrick, 2008 ▶); program(s) used to refine structure: *SHELXL97* (Sheldrick, 2008 ▶); molecular graphics: *Stereochemical Workstation Operation Manual* (Siemens, 1989 ▶); software used to prepare material for publication: *SHELXL97*.

## Supplementary Material

Crystal structure: contains datablocks I, global. DOI: 10.1107/S1600536809000713/lx2085sup1.cif

Structure factors: contains datablocks I. DOI: 10.1107/S1600536809000713/lx2085Isup2.hkl

Additional supplementary materials:  crystallographic information; 3D view; checkCIF report

## Figures and Tables

**Table 1:** Hydrogen-bond geometry (Å, °)

*D*—H⋯*A*	*D*—H	H⋯*A*	*D*⋯*A*	*D*—H⋯*A*
N1*B*—H1*B*⋯Cl1	0.88 (3)	2.28 (4)	3.126 (2)	161 (3)
N1*A*—H1*A*⋯Cl2^i^	0.84 (3)	2.41 (3)	3.127 (2)	145 (3)
N31*A*—H31*A*⋯Cl2^ii^	0.90 (4)	2.51 (4)	3.323 (3)	151 (3)
N31*A*—H31*B*⋯Cl3	0.96 (4)	2.33 (4)	3.267 (3)	167 (3)
C6*B*—H6*B*⋯Cl4	0.95	2.71	3.647 (3)	171

Symmetry codes: (i) 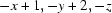; (ii) 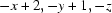.

**Fig. 1.**
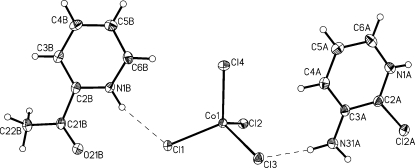
Anisotropic ellipsoid representation of compound 1 together with atom labelling scheme (Siemens, 1989). The ellipsoids are drawn at 50% probability level, hydrogen atoms are depicted as spheres with arbitrary radii. Hydrogen bonds are drawn as dashed lines.

**Fig. 2.**
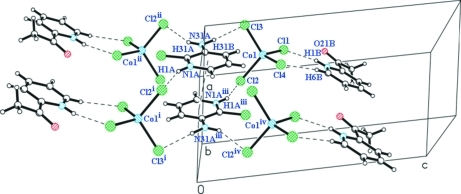
The fragment of the crystal packing of complex 1. Hydrogen bonds and π-π interactions are shown as dashed lines. Symmetry codes: (i) -x+1, -y+2, -z; (ii) -x+2, -y+1, -z; (iii) -x+1, -y+2, -z; (iv) x-1, y+1 , z.]

## supplementary crystallographic information

### Comment

Schiff bases are often employed as ligands in the metal ion - directed assembly of coordination architectures (Radecka-Paryzek *et al.*, 2005). Such complexes are used as luminescent probes in the visible and near-IR spectral domains (Yam *et al.*, 1999), as precursors for doped materials where metal centers must be implemented at a fixed distance and as catalysts for specific DNA (Mukherjee *et al.*, 2005) and RNA (Häner & Hall, 1999) cleavage. In the course of our studies of Schiff base metal complexes with novel chemical properties we have accidentally synthesized the interesting example of three-component complex with CoCl_4_ dianion and two different cations: 3-amino-chloropyridinium (*a*) and 2-acetylpyridinium (*b*) (Scheme & Fig. 1).

Both cations are planar within the experimental error; the maximum deviation from the least-squares planes are as small as 0.006 (2)Å in (*a*) and 0.002 (2)Å in (*b*). In the latter case the plane of acetyl group makes a dihedral angle of 11.0 (2)° with the ring plane. In the crystal structure, two motifs involving the (*a*) cations, *R*^4^_4_(12) and *R*^4^_4_(18, act together to make the double chain of these cations and dianions along [110] direction. The *R*^4^_4_(18) motif is additionally strengthened by the π-π stacking of pyridinium rings. The distance between the exactly parallel least-squares planes is 3.389 (3) Å, with relatively small offset of only 0.708 Å. The second kind of cations, (*b*) are joined - by means of the N—H···Cl hydrogen bonds - to the chain, on its both sides (Fig. 2). Additionally, relatively short and linear C—H···Cl hydrogen bond is accepted by the Cl4 atom, not involved in any N—H···Cl interactions.

The Co is tetrahedrally coordinated in the anions (Fig. 1); the distortion from the ideal geometry is small. The angles are close to the ideal values {106.68 (3) - 112.35 (3)°}. The differences in the Co—Cl bond lengths correlate with the number of hydrogen bonds accepted by the Cl atom: Co—Cl2 bond is the longest {2.2893 (7) Å; Cl2 accepts two h.b.'s}, Co—Cl1 and Co—Cl3 have similar, intermediate lengths of 2.2751 (7)Å and 2.2771 (7) Å, and Cl4, which accepts only C—H···Cl hydrogen bonds, makes the shortest Co—Cl bond of 2.2593 (7) Å.

### Experimental

To a mixture of cobalt chloride hexahydrate (18.2 mg; 0.08 mmol) and 2-acetylpyridine (9.4 mg; 0.08.m mol) in acetonitrile (20 cm^3^), 3-amino-2-chloropyridine (0.01 g; 0.08 mmol) in acetonitrile (10 cm^3^) was added dropwise with stirring. The reaction mixture was stirred for 24 h, at room temperature. The green crystals were obtained by slow diffusion of chloroform to the acetonitrile solution.

### Refinement

Hydrogen atoms from N—H groups were located in difference Fourier maps and isotropically refined; other H atoms were located geometrically and refined as the 'riding model' with *U*_iso_'s set at 1.2 (1.4 for methyl group) times *U*_eq_'s of appropriate oxygen atoms.

### Crystal data

**Table:**

(C_5_H_6_ClN_2_)(C_7_H_8_NO)[CoCl_4_]	*Z* = 2
*M**_r_* = 452.44	*F*(000) = 454
Triclinic, *P*1	*D*_x_ = 1.700 Mg m^−^^3^
Hall symbol: -P 1	Mo *K*α radiation, λ = 0.71073 Å
*a* = 7.3255 (5) Å	Cell parameters from 7316 reflections
*b* = 8.3188 (5) Å	θ = 3–25°
*c* = 16.2657 (11) Å	µ = 1.73 mm^−^^1^
α = 89.114 (5)°	*T* = 100 K
β = 82.806 (5)°	Plate, blue
γ = 64.145 (6)°	0.4 × 0.15 × 0.1 mm
*V* = 884.13 (10) Å^3^	

### Data collection

**Table:**

Kuma KM-4-CCD four-circle diffractometer	3798 independent reflections
Radiation source: fine-focus sealed tube	3470 reflections with *I* > 2σ(*I*)
graphite	*R*_int_ = 0.019
Detector resolution: 8.1929 pixels mm^-1^	θ_max_ = 27.0°, θ_min_ = 2.7°
ω scans	*h* = −9→9
Absorption correction: multi-scan (*CrysAlis RED*; Oxford Diffraction, 2007)	*k* = −10→10
*T*_min_ = 0.616, *T*_max_ = 0.841	*l* = −20→19
10954 measured reflections	

### Refinement

**Table:**

Refinement on *F*^2^	Primary atom site location: structure-invariant direct methods
Least-squares matrix: full	Secondary atom site location: difference Fourier map
*R*[*F*^2^ > 2σ(*F*^2^)] = 0.031	Hydrogen site location: difference Fourier map
*wR*(*F*^2^) = 0.065	H atoms treated by a mixture of independent and constrained refinement
*S* = 1.24	*w* = 1/[σ^2^(*F*_o_^2^) + (0.0083*P*)^2^ + 1.4737*P*] where *P* = (*F*_o_^2^ + 2*F*_c_^2^)/3
3798 reflections	(Δ/σ)_max_ < 0.001
216 parameters	Δρ_max_ = 0.70 e Å^−^^3^
0 restraints	Δρ_min_ = −0.36 e Å^−^^3^

### Special details

**Table:**

Geometry. All e.s.d.'s (except the e.s.d. in the dihedral angle between two l.s. planes) are estimated using the full covariance matrix. The cell e.s.d.'s are taken into account individually in the estimation of e.s.d.'s in distances, angles and torsion angles; correlations between e.s.d.'s in cell parameters are only used when they are defined by crystal symmetry. An approximate (isotropic) treatment of cell e.s.d.'s is used for estimating e.s.d.'s involving l.s. planes.
Refinement. Refinement of *F*^2^ against ALL reflections. The weighted *R*-factor *wR* and goodness of fit *S* are based on *F*^2^, conventional *R*-factors *R* are based on *F*, with *F* set to zero for negative *F*^2^. The threshold expression of *F*^2^ > σ(*F*^2^) is used only for calculating *R*-factors(gt) *etc*. and is not relevant to the choice of reflections for refinement. *R*-factors based on *F*^2^ are statistically about twice as large as those based on *F*, and *R*- factors based on ALL data will be even larger.

### Fractional atomic coordinates and isotropic or equivalent isotropic displacement parameters (Å^2^)

**Table:**

	*x*	*y*	*z*	*U*_iso_*/*U*_eq_	
Co1	0.88393 (5)	0.59259 (4)	0.24288 (2)	0.01220 (9)	
Cl1	1.00305 (9)	0.37850 (8)	0.33709 (4)	0.01584 (13)	
Cl2	0.70499 (9)	0.51487 (8)	0.15843 (4)	0.01614 (13)	
Cl3	1.15608 (9)	0.60998 (8)	0.16588 (4)	0.01751 (13)	
Cl4	0.66564 (10)	0.86213 (8)	0.30354 (4)	0.01991 (14)	
N1A	0.5900 (3)	1.1877 (3)	−0.05439 (15)	0.0177 (5)	
H1A	0.542 (5)	1.229 (4)	−0.098 (2)	0.026 (9)*	
C2A	0.7364 (4)	1.0189 (3)	−0.06482 (16)	0.0175 (5)	
Cl2A	0.82058 (10)	0.92797 (9)	−0.16337 (4)	0.02218 (15)	
C3A	0.8134 (4)	0.9213 (3)	0.00390 (16)	0.0161 (5)	
N31A	0.9566 (4)	0.7490 (3)	−0.00534 (16)	0.0236 (5)	
H31A	1.011 (5)	0.705 (4)	−0.058 (2)	0.033 (9)*	
H31B	1.015 (5)	0.690 (4)	0.042 (2)	0.032 (9)*	
C4A	0.7311 (4)	1.0089 (4)	0.08237 (17)	0.0186 (5)	
H4A	0.7798	0.9479	0.1307	0.022*	
C5A	0.5794 (4)	1.1834 (4)	0.08995 (17)	0.0209 (6)	
H5A	0.5254	1.2408	0.1434	0.025*	
C6A	0.5065 (4)	1.2742 (4)	0.02109 (18)	0.0198 (6)	
H6A	0.4014	1.3933	0.0259	0.024*	
N1B	0.7520 (3)	0.5002 (3)	0.51335 (13)	0.0148 (4)	
H1B	0.844 (5)	0.447 (4)	0.470 (2)	0.032 (9)*	
C2B	0.7401 (4)	0.4099 (3)	0.58207 (15)	0.0148 (5)	
C21B	0.9013 (4)	0.2182 (3)	0.57886 (16)	0.0162 (5)	
O21B	1.0417 (3)	0.1707 (3)	0.52259 (12)	0.0222 (4)	
C22B	0.8761 (4)	0.1020 (4)	0.64563 (17)	0.0210 (6)	
H22A	0.9764	−0.0222	0.6320	0.029*	
H22B	0.7376	0.1102	0.6503	0.029*	
H22C	0.8978	0.1416	0.6985	0.029*	
C3B	0.5901 (4)	0.4969 (4)	0.64742 (16)	0.0176 (5)	
H3B	0.5773	0.4356	0.6959	0.021*	
C4B	0.4563 (4)	0.6782 (4)	0.64097 (17)	0.0201 (6)	
H4B	0.3530	0.7407	0.6857	0.024*	
C5B	0.4745 (4)	0.7655 (4)	0.56984 (17)	0.0198 (6)	
H5B	0.3839	0.8878	0.5653	0.024*	
C6B	0.6263 (4)	0.6729 (3)	0.50503 (17)	0.0179 (5)	
H6B	0.6410	0.7308	0.4556	0.022*	

### Atomic displacement parameters (Å^2^)

**Table:**

	*U*^11^	*U*^22^	*U*^33^	*U*^12^	*U*^13^	*U*^23^
Co1	0.01261 (16)	0.01326 (16)	0.01084 (17)	−0.00584 (13)	−0.00127 (12)	0.00103 (13)
Cl1	0.0180 (3)	0.0160 (3)	0.0126 (3)	−0.0066 (2)	−0.0024 (2)	0.0034 (2)
Cl2	0.0149 (3)	0.0185 (3)	0.0152 (3)	−0.0069 (2)	−0.0039 (2)	−0.0007 (2)
Cl3	0.0151 (3)	0.0224 (3)	0.0162 (3)	−0.0097 (2)	−0.0007 (2)	0.0032 (2)
Cl4	0.0213 (3)	0.0151 (3)	0.0190 (3)	−0.0047 (2)	0.0003 (2)	−0.0023 (2)
N1A	0.0163 (11)	0.0158 (11)	0.0217 (12)	−0.0073 (9)	−0.0041 (9)	0.0036 (9)
C2A	0.0176 (12)	0.0193 (13)	0.0167 (13)	−0.0099 (10)	0.0010 (10)	0.0011 (10)
Cl2A	0.0260 (3)	0.0232 (3)	0.0140 (3)	−0.0083 (3)	−0.0004 (2)	0.0017 (2)
C3A	0.0156 (12)	0.0185 (13)	0.0174 (13)	−0.0105 (10)	−0.0020 (10)	0.0037 (10)
N31A	0.0246 (12)	0.0212 (12)	0.0157 (12)	−0.0018 (10)	−0.0010 (10)	0.0022 (10)
C4A	0.0201 (13)	0.0201 (13)	0.0172 (13)	−0.0106 (11)	−0.0019 (10)	0.0023 (11)
C5A	0.0221 (13)	0.0223 (14)	0.0211 (14)	−0.0132 (11)	0.0012 (11)	−0.0038 (11)
C6A	0.0190 (13)	0.0161 (13)	0.0300 (15)	−0.0122 (11)	−0.0072 (11)	0.0051 (11)
N1B	0.0162 (10)	0.0166 (11)	0.0122 (10)	−0.0077 (9)	−0.0016 (8)	−0.0002 (9)
C2B	0.0160 (12)	0.0197 (13)	0.0131 (12)	−0.0114 (10)	−0.0038 (10)	0.0004 (10)
C21B	0.0184 (12)	0.0193 (13)	0.0140 (13)	−0.0102 (10)	−0.0051 (10)	0.0014 (10)
O21B	0.0212 (10)	0.0225 (10)	0.0173 (10)	−0.0051 (8)	−0.0007 (8)	0.0025 (8)
C22B	0.0244 (14)	0.0222 (14)	0.0183 (13)	−0.0117 (11)	−0.0048 (11)	0.0063 (11)
C3B	0.0177 (12)	0.0256 (14)	0.0138 (12)	−0.0133 (11)	−0.0021 (10)	0.0009 (11)
C4B	0.0167 (12)	0.0237 (14)	0.0195 (14)	−0.0088 (11)	0.0001 (10)	−0.0066 (11)
C5B	0.0196 (13)	0.0175 (13)	0.0231 (14)	−0.0083 (11)	−0.0046 (11)	−0.0021 (11)
C6B	0.0221 (13)	0.0176 (13)	0.0175 (13)	−0.0112 (11)	−0.0056 (10)	0.0039 (10)

### Geometric parameters (Å, °)

**Table:**

Co1—Cl4	2.2593 (7)	N1B—C6B	1.342 (3)
Co1—Cl1	2.2751 (7)	N1B—C2B	1.352 (3)
Co1—Cl3	2.2771 (7)	N1B—H1B	0.88 (3)
Co1—Cl2	2.2893 (7)	C2B—C3B	1.378 (4)
N1A—C2A	1.341 (3)	C2B—C21B	1.512 (4)
N1A—C6A	1.362 (4)	C21B—O21B	1.214 (3)
N1A—H1A	0.84 (3)	C21B—C22B	1.490 (4)
C2A—C3A	1.399 (4)	C22B—H22A	0.9800
C2A—Cl2A	1.705 (3)	C22B—H22B	0.9800
C3A—N31A	1.354 (3)	C22B—H22C	0.9800
C3A—C4A	1.406 (4)	C3B—C4B	1.406 (4)
N31A—H31A	0.90 (4)	C3B—H3B	0.9500
N31A—H31B	0.96 (4)	C4B—C5B	1.379 (4)
C4A—C5A	1.386 (4)	C4B—H4B	0.9500
C4A—H4A	0.9500	C5B—C6B	1.388 (4)
C5A—C6A	1.372 (4)	C5B—H5B	0.9500
C5A—H5A	0.9500	C6B—H6B	0.9500
C6A—H6A	0.9500		
			
Cl4—Co1—Cl1	112.35 (3)	C6B—N1B—C2B	123.6 (2)
Cl4—Co1—Cl3	110.42 (3)	C6B—N1B—H1B	116 (2)
Cl1—Co1—Cl3	108.54 (3)	C2B—N1B—H1B	121 (2)
Cl4—Co1—Cl2	106.68 (3)	N1B—C2B—C3B	119.1 (2)
Cl1—Co1—Cl2	109.10 (3)	N1B—C2B—C21B	114.6 (2)
Cl3—Co1—Cl2	109.72 (3)	C3B—C2B—C21B	126.3 (2)
C2A—N1A—C6A	123.7 (2)	O21B—C21B—C22B	124.9 (2)
C2A—N1A—H1A	113 (2)	O21B—C21B—C2B	117.8 (2)
C6A—N1A—H1A	123 (2)	C22B—C21B—C2B	117.3 (2)
N1A—C2A—C3A	120.3 (2)	C21B—C22B—H22A	109.5
N1A—C2A—Cl2A	118.0 (2)	C21B—C22B—H22B	109.5
C3A—C2A—Cl2A	121.7 (2)	H22A—C22B—H22B	109.5
N31A—C3A—C2A	121.0 (2)	C21B—C22B—H22C	109.5
N31A—C3A—C4A	122.0 (2)	H22A—C22B—H22C	109.5
C2A—C3A—C4A	116.9 (2)	H22B—C22B—H22C	109.5
C3A—N31A—H31A	117 (2)	C2B—C3B—C4B	118.8 (2)
C3A—N31A—H31B	119 (2)	C2B—C3B—H3B	120.6
H31A—N31A—H31B	123 (3)	C4B—C3B—H3B	120.6
C5A—C4A—C3A	120.7 (2)	C5B—C4B—C3B	120.2 (2)
C5A—C4A—H4A	119.7	C5B—C4B—H4B	119.9
C3A—C4A—H4A	119.7	C3B—C4B—H4B	119.9
C6A—C5A—C4A	120.7 (3)	C4B—C5B—C6B	119.4 (2)
C6A—C5A—H5A	119.6	C4B—C5B—H5B	120.3
C4A—C5A—H5A	119.6	C6B—C5B—H5B	120.3
N1A—C6A—C5A	117.7 (2)	N1B—C6B—C5B	118.9 (2)
N1A—C6A—H6A	121.2	N1B—C6B—H6B	120.5
C5A—C6A—H6A	121.2	C5B—C6B—H6B	120.5
			
C6A—N1A—C2A—C3A	−0.5 (4)	C6B—N1B—C2B—C21B	−178.2 (2)
C6A—N1A—C2A—Cl2A	178.5 (2)	N1B—C2B—C21B—O21B	10.7 (3)
N1A—C2A—C3A—N31A	177.9 (2)	C3B—C2B—C21B—O21B	−168.3 (2)
Cl2A—C2A—C3A—N31A	−1.0 (4)	N1B—C2B—C21B—C22B	−169.7 (2)
N1A—C2A—C3A—C4A	−0.3 (4)	C3B—C2B—C21B—C22B	11.3 (4)
Cl2A—C2A—C3A—C4A	−179.19 (19)	N1B—C2B—C3B—C4B	−1.1 (4)
N31A—C3A—C4A—C5A	−177.7 (3)	C21B—C2B—C3B—C4B	177.9 (2)
C2A—C3A—C4A—C5A	0.5 (4)	C2B—C3B—C4B—C5B	0.8 (4)
C3A—C4A—C5A—C6A	0.0 (4)	C3B—C4B—C5B—C6B	−0.2 (4)
C2A—N1A—C6A—C5A	1.0 (4)	C2B—N1B—C6B—C5B	−0.3 (4)
C4A—C5A—C6A—N1A	−0.8 (4)	C4B—C5B—C6B—N1B	−0.1 (4)
C6B—N1B—C2B—C3B	0.9 (4)		

### Hydrogen-bond geometry (Å, °)

**Table:**

*D*—H···*A*	*D*—H	H···*A*	*D*···*A*	*D*—H···*A*
N1B—H1B···Cl1	0.88 (3)	2.28 (4)	3.126 (2)	161 (3)
N1A—H1A···Cl2^i^	0.84 (3)	2.41 (3)	3.127 (2)	145 (3)
N31A—H31A···Cl2^ii^	0.90 (4)	2.51 (4)	3.323 (3)	151 (3)
N31A—H31B···Cl3	0.96 (4)	2.33 (4)	3.267 (3)	167 (3)
C6B—H6B···Cl4	0.95	2.71	3.647 (3)	171

Symmetry codes: (i) −*x*+1, −*y*+2, −*z*; (ii) −*x*+2, −*y*+1, −*z*.

## References

[bb1] Byrn, M. P., Curtis, C. J., Hsiou, Y., Khan, S. I., Sawin, P. A., Tendick, S. K., Terzis, A. & Strouse, C. E. (1993). *J. Am. Chem. Soc.* **115**, 9480–9497.

[bb2] Hamed, K., Samah, A. & Mohamed, R. (2007). *Acta Cryst.* E**63**, o2896.

[bb3] Häner, R. & Hall, J. (1999). *Antisense Nucleic Acid Drug Dev.* **7**, 423–430. 10.1089/oli.1.1997.7.423 9303194

[bb4] Husak, M. (1996). Private communication (refcode NABLIL). CCDC, Cambridge, England.

[bb5] Laurent, A. (1966). *Acta Cryst.* **21**, 710–715.

[bb6] Li, W., Tong, M.-L., Chen, X.-M., Yuan, J.-X. & Hu, M.-L. (2002). *Acta Cryst.* E**58**, m203–m205.

[bb7] Mukherjee, A., Dhar, S., Nethaji, M. & Chakravarty, A. R. (2005). *Dalton Trans.* pp. 349–353. 10.1039/b415864d 15616725

[bb8] Oxford Diffraction (2007). *CrysAlis CCD* and *CrysAlis RED* Oxford Diffraction Ltd, Oxfordshire, England.

[bb9] Radecka-Paryzek, W., Patroniak, V. & Lisowski, J. (2005). *Coord. Chem. Rev.* **249**, 2156–2175.

[bb10] Saha, B. K., Nangia, A. & Nicoud, J.-F. (2006). *Cryst. Growth Des.* **6**, 1278–1281.

[bb11] Sheldrick, G. M. (2008). *Acta Cryst.* A**64**, 112–122. 10.1107/S0108767307043930 18156677

[bb12] Siemens (1989). *Stereochemical Workstation Operation Manual* Siemens Analytical X-ray Instruments Inc., Madison, Wisconsin, USA.

[bb13] Tong, M.-L., Chen, X.-M. & Ng, S. W. (2002). *Acta Cryst.* C**58**, m481–m482. 10.1107/S0108270102013872 12205377

[bb14] Yam, V. & Lo, K. K.-W. (1999). *Coord. Chem. Rev.* **184**, 157–240.

